# Draft Genome Sequences of Pseudomonas spp. Isolated from Berry Surfaces in Commercial Cranberry Bogs in Massachusetts, USA

**DOI:** 10.1128/MRA.00204-21

**Published:** 2021-07-08

**Authors:** Marit H. Koszewski, Sheyda Motevalli, Scott D. Soby

**Affiliations:** a Biomedical Sciences Program, College of Graduate Studies, Midwestern University, Glendale, Arizona, USA; b College of Veterinary Medicine, Midwestern University, Glendale, Arizona, USA; University of Arizona

## Abstract

The surfaces of plants are colonized by a rich diversity of microbes but are largely unexplored. Here, we present the draft genome sequences of five Pseudomonas spp. isolated from cultivated cranberry fruit surfaces. Although the isolates represent four different species, their genomes all contain conserved iron sequestration and uptake genes.

## ANNOUNCEMENT

The genus Pseudomonas (*Gammaproteobacteria*) is widely recognized as being among the most diverse and ubiquitous bacterial taxa, with 242 currently validated species (https://lpsn.dsmz.de/genus/pseudomonas). Members of the genus include human, animal, and plant pathogens (1–4), inhabit diverse habitats (5–8), and play important roles in plant growth, development, and protection from disease (9–11). We recently demonstrated that Pseudomonas spp. isolated from cranberry plants produce volatile organic compounds that inhibit the growth of several types of plant-associated fungi and Phytophthora cinnamomi (12). Despite their ubiquity and importance, little is known about the Pseudomonas spp. that inhabit the surfaces of plant organs or what their functional roles are in those niches. Recently, we explored the bacteria colonizing the surfaces of cranberry plants (Vaccinium macrocarpon Ait.) (13–17). The ability to analyze and compare the genomes of these nonpathogenic commensal bacteria is providing new insights into the relationships between plants and their microbiomes and may yield new methods for controlling fungal infections that lead to crop loss.

**FIG 1:**
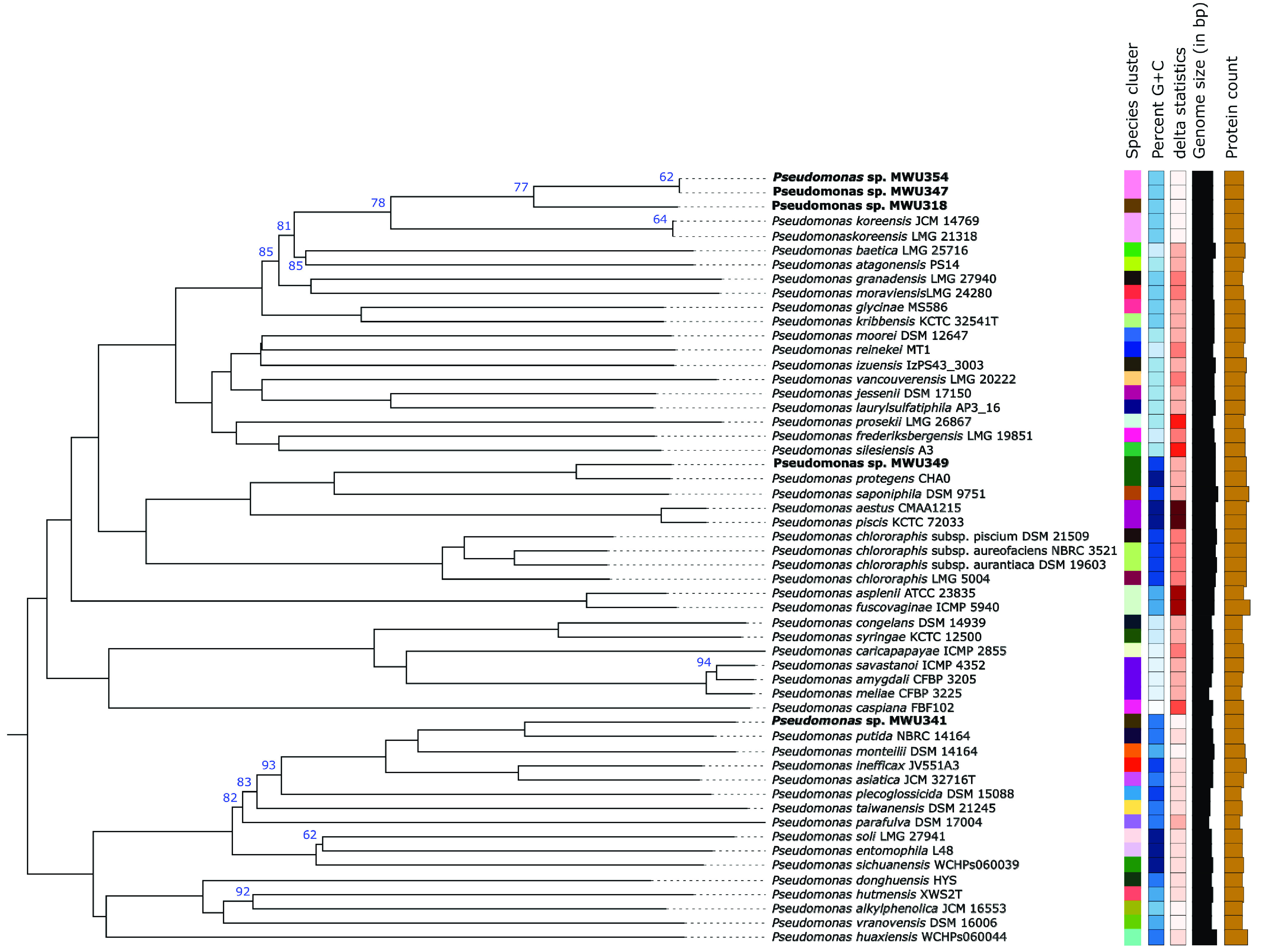
Genome BLAST Distance Phylogeny (GBDP) tree. A phylogenetic tree was constructed with the Type (Strain) Genome Server (19), which produces a GBDP tree by approximating intergenomic relatedness using the MASH algorithm among all type strain genomes in the TYGS database and by extracting and comparing 16S rRNA gene sequences with 12,670 type strains using BLAST as a proxy to identify the 50 closest type strains to calculate precise distances. The tree itself was constructed using FastME version 2.1.4 to infer a balanced minimum evolution tree with branch support (23). The tree represents only the Pseudomonas spp. most closely related to the described isolates. Bootstrap support values are shown at the nodes. Nodes without values have 100% bootstrap support. Isolates described in the text are in bold font. Isolates MWU347 and MWU354 are the same species, MWU347/MWU354, MWU318, and MWU341 represent new species, and MWU349 is P. protegens.

Bacteria were isolated from berries that were aseptically collected in August 2010 from commercial cranberry bogs. Berries were vortexed in sterile water, and the water was plated on King’s medium B (KMB) agar containing 50 μg ml^−1^ each of cycloheximide and ampicillin. Single colonies that fluoresced under long-wave UV light were transferred to fresh medium, colony purified 3 times, and stored at −80°C in 34% glycerol. Isolates were placed in the genus Pseudomonas by phenotype and 16S rRNA gene sequences amplified with 27F and 1525R primers using BLAST (18). Taxonomic placement was verified using the Type (Strain) Genome Server (Fig. 1) (19). Isolates were recovered from frozen storage, streaked onto KMB agar, and inoculated into overnight KMB broth cultures for genomic DNA (gDNA) isolation with a DNeasy blood and tissue kit (Qiagen). Genomic DNA libraries (KAPA HyperPlus library preparation kit) were analyzed for fragment size with an Agilent TapeStation and quantified by quantitative PCR (qPCR) (KAPA library quantification kit) with a QuantStudio 5 system (Thermo Fisher Scientific) before sequencing (Illumina MiSeq 2 × 250-bp flow cell). Raw reads were assembled using Unicycler+ with SPAdes and Pilon version 1.23 for polishing within the PATRIC Comprehensive Genome Analysis pipeline version 3.6.8 with default settings (http://patricbrc.org) (20) (Table 1). The compiled genome sequences were annotated using RASTtk (21).

**TABLE 1:** Genome features of Pseudomonas sp. isolates

Isolate	Assigned taxon	Collection site	Genome size (bp)	No. of contigs	*N*_50_ contig size (bp)	Coverage (×)	G+C content (%)	BioSample no.	GenBank accession no.	SRA accession no.	No. of coding sequences	No. of siderophore-related genes
MWU318	*Nov. sp.*	West Wareham, MA	6,035,781	33	489,847	174	60.3	SAMN17284639	JAESJL000000000	SRX10176289	5,150	17
MWU341	P. protegens	Carver, MA	5,851,990	72	336,634	182	62.4	SAMN17284726	JAERIH000000000	SRX10166717	5,460	21
MWU347	*Nov. sp.*	East Wareham, MA	6,137,901	45	556,804	87	60.3	SAMN17284874	JAFGZB000000000	SRX10299995	5,581	17
MWU349	*Nov. sp.*	East Wareham, MA	6,715,860	18	906,199	228	63.3	SAMN17284895	JAFEVP000000000	SRX10166718	6,199	27
MWU354	*Nov. sp.*	East Wareham, MA	6,137,275	46	531,002	92	60.3	SAMN17284896	JAFGZC000000000	SRX10299996	5,572	17

Isolate MWU349 is Pseudomonas protegens *sensu lato*, but the other isolates were not assigned to a specific taxon (Fig. 1). MWU354 and MWU347 are members of the same *nova species* but are not clonal isolates. As an indication of the importance of iron sequestration in the berry surface microenvironment (22), each of the isolates has multiple siderophore-related genes, including nonribosomal peptide synthases for the production of pyoverdine-like siderophores. TonB-dependent hemin receptors, iron siderophore sensor proteins, pyoverdine chromophore precursor synthase PvdL, and the iron dicitrate transport protein FecA are conserved across all of the isolates.

### Data availability.

The Pseudomonas sp. strain MWU318, MWU341, MWU347, MWU349, and MWU354 genome sequences have been deposited in GenBank under BioProject number PRJNA691338. This whole-genome shotgun project has been deposited at DDBJ/ENA/GenBank under the whole-genome sequence (WGS) and SRA accession numbers in Table 1.
